# Variation in Fetal Outcome, Viral Load and ORF5 Sequence Mutations in a Large Scale Study of Phenotypic Responses to Late Gestation Exposure to Type 2 Porcine Reproductive and Respiratory Syndrome Virus

**DOI:** 10.1371/journal.pone.0096104

**Published:** 2014-04-22

**Authors:** Andrea Ladinig, Jamie Wilkinson, Carolyn Ashley, Susan E. Detmer, Joan K. Lunney, Graham Plastow, John C. S. Harding

**Affiliations:** 1 Department of Large Animal Clinical Sciences, Western College of Veterinary Medicine, University of Saskatchewan, Saskatoon, Saskatchewan, Canada; 2 Department of Agricultural, Food, and Nutritional Science, Faculty of Agricultural, Life and Environmental Sciences, University of Alberta, Edmonton, Alberta, Canada; 3 Department of Veterinary Pathology, Western College of Veterinary Medicine, University of Saskatchewan, Saskatoon, Saskatchewan, Canada; 4 Animal Parasitic Diseases Laboratory, Beltsville Agricultural Research Center, Agricultural Research Service, U.S. Department of Agriculture, Beltsville, Maryland, United States of America; Virginia Polytechnic Institute and State University, United States of America

## Abstract

In spite of extensive research, the mechanisms of reproductive disease associated with Porcine Reproductive and Respiratory Syndrome virus (PRRSv) are still poorly understood. The objectives of this large scale study were to evaluate associations between viral load and fetal preservation, determine the impact of type 2 PRRSv on fetal weights, and investigate changes in ORF5 PRRSv genome in dams and fetuses during a 21-day period following challenge. At gestation day 85 (±1), 114 gilts were experimentally infected with type 2 PRRSv, while 19 gilts served as reference controls. At necropsy, fetuses were categorized according to their preservation status and tissue samples were collected. PRRSv RNA concentrations were measured in gilt serum collected on days 0, 2, 6, and 21 post-infection, as well as in gilt and fetal tissues collected at termination. Fetal mortality was 41±22.8% in PRRS infected litters. Dead fetuses appeared to cluster in some litters but appeared solitary or random in others. Nine percent of surviving piglets were meconium-stained. PRRSv RNA concentration in fetal thymus, fetal serum and endometrium differed significantly across preservation category and was greatest in tissues of meconium-stained fetuses. This, together with the virtual absence of meconium staining in non-infected litters indicates it is an early pathological condition of reproductive PRRS. Viral load in fetal thymus and in fetal serum was positively associated with viral load in endometrium, suggesting the virus exploits dynamic linkages between individual maternal-fetal compartments. Point mutations in ORF5 sequences from gilts and fetuses were randomly located in 20 positions in ORF5, but neither nucleotide nor amino acid substitutions were associated with fetal preservation. PRRSv infection decreased the weights of viable fetuses by approximately 17%. The considerable variation in gilt and fetal outcomes provides tremendous opportunity for more detailed investigations of potential mechanisms and single nucleotide polymorphisms associated with fetal death.

## Introduction

The Porcine Reproductive and Respiratory Syndrome virus (PRRSv) causes reproductive failure in gilts and sows in early and late gestation. While in early gestation the virus can cause embryonic death [1], [2], the clinical manifestation of PRRSv mainly occurs in late gestation and is characterized by abortions, early farrowings, fetal death, and the birth of weak, congenitally infected piglets resulting in elevated pre-weaning mortality [3], [4]. Although fetuses are susceptible to PRRSv at any stage of gestation upon direct intra-fetal inoculation [5], [6], transplacental PRRSv infection mainly occurs in late gestation [5], [7]. Underlying mechanisms of PRRSv-induced reproductive failure occurring in late gestation are poorly understood. It has been suggested that fetal death may not be a direct result of PRRSv replication in fetal tissues since severe microscopic lesions are not observed in infected fetuses [8], [9]. It has recently been shown that the number of sialoadhesin positive (Sn+)/CD163+ macrophages in endometrium and placenta [10], and virus replication in fetal implantation sites which causes apoptosis of infected and surrounding cells [11] play a role in fetal death. However, the exact mechanisms by which PRRSv transmits from the dam to her fetuses have yet to be determined [12]. Once infected, PRRSv can be detected systemically in several fetal tissues including lung, liver, spleen, heart and kidney, but the virus is most consistently found in lymphatic tissues with the fetal thymus being the primary site of virus replication [13], [14].

During replication *in vivo* and in cell culture, mutations appear in the PRRSv genome leading to the existence of closely related viral particles called quasispecies, commonly observed in RNA viruses [14], [15]. The detection of mutations, most commonly performed by sequencing the hypervariable region of ORF5, allows for the monitoring of PRRSv evolution within a population [15], [16]. It has been suggested that fetal infection might be a potential source of PRRSv diversity, and that fetuses might be capable of selecting for a particular virus population [14].

A large scale, multi-institutional project aimed at finding phenotypic and genotypic predictors of PRRSv resistance in pregnant gilts was undertaken at the University of Saskatchewan, Saskatoon, Canada in 2012. While analyses of an extensive and complex dataset are ongoing, the results presented herein describe novel aspects of the phenotypic responses in dam and fetuses following PRRSv challenge in the third trimester of pregnancy. The objectives of the present study were to: 1) investigate relationships between viral load in dam and fetus, and fetal preservation category in a large number of infected gilts; 2) determine the impact of PRRSv on fetal weight and investigate associations with viral load; 3) investigate changes in the ORF5 sequence in dams and fetuses during a 21-day period following challenge.

## Materials and Methods

### Ethics Statement

Inoculation of gilts or sows in the last trimester of gestation is a widely accepted and commonly used model of studying reproductive PRRS [11], [13], [14], [17]–[19]. Although we recognize that some fetuses die after inoculation, no alternative models are available to study the reproductive effects of PRRSv. Fetal death can occur any time after inoculation and it is not possible to predict if and when it will occur on any individual animal. Monitoring fetuses for stress and discomfort is not feasible in a litter bearing species like swine. A humane intervention point (HIP) checklist was developed and approved specifically for this project. Gilts were monitored according to that HIP checklist twice daily. Clinical signs in gilts were mild or absent, so medical interventions such as analgesics or anesthetics were not justified. Animal numbers were carefully considered. The number of control animals was reduced to a minimum to provide baseline data and the number of inoculated gilts was selected to enable both deep phenotyping and genotyping of gilts and fetuses. Given that fetal death was an outcome, the experimental protocol was considered carefully before approval by the University of Saskatchewan’s Animal Research Ethics Board. It adhered to the Canadian Council on Animal Care guidelines for humane animal use (permit #20110102).

### Animals

Purebred Landrace gilts (n = 133) were obtained from a high-health nucleus herd free of PRRSv, *Mycoplasma hyopneumoniae* and *Actinobacillus pleuropneumoniae* based on absence of clinical signs and routine serologic monitoring. Gilts were vaccinated against porcine parvovirus (PPV), erysipelas, *Leptospira* sp. (Farrowsure Gold B, Zoetis, Canada, Kirkland, QC) twice prior to breeding, porcine circovirus type 2 (PCV2; Circoflex, Boehringer-Ingelheim (Canada) Ltd. Burlington, ON) at 3 weeks of age, and *Haemophilus parasuis* (Suvaxyn HPS, Zoetis) at 9 and 22 weeks of age. One hundred and thirty-three gilts were selected at approximately 150 days of age in bi-weekly repetitions (reps) (median 11, range 3–15 gilts/rep, total of 12 reps). Gilts were estrus-stimulated by direct daily boar contact and after each gilt had shown at least one estrus, they were moved into individual crates and synchronized by daily oral administration of 17.6 mg altrenogest (8 mL Regu-Mate 0.22% Solution, Merck Animal Health, Kirkland, QC) for a total of 18 days. Thirty-six hours after the last Regu-Mate dose, 800 international units (IU) pregnant mare serum gonadotropin (4 mL Folligon, Merck Animal Health) was administered intramuscularly (IM). Gilts were artificially inseminated using homospermic semen from one of 24 Yorkshire boars. Gilts that failed to conceive were re-inseminated on their next estrus. Pregnancy was confirmed ultrasonically, and pregnant gilts were housed in gestation stalls until gestation day 80 (±1) when they were transported to a biosafety level 2 (BSL2) animal care facility at the University of Saskatchewan. Prior to transportation, blood was drawn from the jugular vein to confirm PRRSv negativity by ELISA (IDEXX PRRS X3 Ab test, IDEXX laboratories, Inc., Maine, US) and PCR (Tetracore PRRS real-time PCR kit, Tetracore, Inc., Rockville, US). In BSL2, gilts were randomly allocated to a PRRSv-challenged or reference control group. These two groups were placed in separate rooms, with gilts housed in individual crates. As the primary objective of the experiment was to investigate variation in severity amongst PRRSv-challenged animals, an unbalanced experimental design was intentionally used with a goal of including 1–2 control gilts per bi-weekly replicate. Gilts had free access to water and were fed a standard wheat/barley-based gestation diet, 2.5 kg once daily, throughout gestation.

### PRRSv Isolate

NVSL 97–7895 (generously provided by R. Rowland, Kansas State University) [20] (GenBank Accession No. AF325691) was propagated in MARC-145 cells (generously provided by S. Carman, Animal Health Laboratory, University of Guelph). Stock viral aliquots (5×10^7^ TCID_50_/mL) in minimum essential medium (MEM) supplemented with 1.6% Penicillin-streptomycin and 7% fetal bovine serum were frozen at −80°C. On each inoculation day, an aliquot was thawed and diluted to a final concentration of 1×10^5^ TCID_50_ in 4 mL MEM.

### Experimental Procedures

After 5 days of acclimation in BSL2, gilts (INOC, n = 114) were inoculated with 1×10^5^ TCID_50_ PRRSv NVSL 97–7895; 2 mL intramuscular (IM) and 1 mL into each nostril. Control gilts (CTRL, n = 19) were similarly mock-infected with MEM. The day of inoculation, gestation day 85 (±1), was considered experimental day 0 (D0). Gilts were observed twice daily for clinical signs and demeanour; rectal temperatures and feed intake were assessed once daily according to the approved HIP checklist. Rectal temperature exceeding 39.5**°**C was considered a fever. Gilts were individually restrained on D0, D2, D6 and D21 using a loop snare and jugular blood was collected into plain coagulation tubes. Serum was harvested, aliquoted and stored at −80**°**C.

At D21 (gestation day 106±1), gilts were sedated with intravenous barbiturate (30 mL Euthanyl Forte supplying 16,200 mg pentobarbital sodium, Vetoquinol, Lavaltrie, QC) and humanely euthanized by cranial captive bolt followed by pithing. The gravid reproductive tract was removed intact, placed in a trough in a linear manner (from left oviduct to right oviduct), and rinsed of maternal blood. The reproductive lymph node (*Lnn. uterini* located in the broad ligament), lung, tracheobronchial lymph node, and tonsil were removed from each gilt and examined for gross pathological changes. Samples of each were collected and immediately frozen at −80**°**C until further processing. To prevent cross contamination necropsy instruments and necropsy surfaces were rinsed and disinfected with Synergize (Pro-AG products ltd., Winnipeg, MB, Canada) for at least 10 min between animals (gilts and fetuses).

### Fetal Assessments

The linearized uterus was carefully opened starting at the tip of each horn. Fetuses were numbered sequentially according to their position within each horn starting with “L1” and “R1” being the fetuses closest to the ovary on the left and right sides respectively. The preservation of each fetus was recorded as: viable (VIA), meconium-stained (MEC), decomposed (DEC), autolysed (AUT), or mummified (MUM) (Table 1). By our definition, MUM had crown rump lengths less than 20 cm, and were considered dead prior to inoculation. Although counted, they were excluded from further analysis. The umbilical cords were clamped, and each fetus was removed with its umbilical cord, placenta and a portion of uterus adjacent to the umbilical stump. Each was placed on a disposable fiberboard tray to prevent contamination with necropsy surfaces. Samples of endometrium (including adherent placental layers) adjacent to the umbilical stump were collected into microcentrifugation tubes, and immediately frozen at −80**°**C. Each fetus was weighed and the sex recorded. The carcass was dissected and samples of thymus, spleen and lung were collected and frozen immediately at −80**°**C. In VIA and MEC fetuses, blood was collected from the axillary artery and serum subsequently separated and stored at −80°C.

**Table 1:** Criteria to define preservation category of fetuses at 21 days post infection.

Preservation category	Appearance of external surfaces	Appearance of internal organs
Viable (VIA)	normal, white to purple with visible hair Engorged, pulsing umbilical cord	normal
Meconium stained (MEC)	alive at termination, covered with inspissated, brownish amniotic fluid Pulsing umbilical cord, ± edema	normal
Decomposed (DEC)	dead, generally white, <50% of surface discoloured, no blood in umbilical cord	liquefied, friable, adhered
Autolysed (AUT)	dead, >50% of surface discoloured	liquefied, friable, adhered
Mummified (MUM)	Small, dehydrated, inspissated remains with crown rump length <20 cm. Excluded from analysis as deemed dead prior to PRRSv inoculation	

Live = VIA plus MEC.

Dead = DEC plus AUT.

### Quantification of PRRSv RNA

PRRSv concentration in D0, D2, D6, and D21 sera (target RNA copies/µL), and in lung, tonsil, reproductive and tracheobronchial lymph nodes (target RNA copies/mg) were measured in all gilts using an in-house quantitative real-time PCR assay (qRT-PCR). Thymus and endometrium (from live and dead fetuses) and fetal serum (from live fetuses only) were also tested from INOC fetuses and a subset of CTRL fetuses (3 randomly selected per litter). RNA was extracted from 140 µl of serum using the QIAamp Viral RNA mini kit (Qiagen Inc., Toronto, ON) or from 10–20 mg of tissue using the RNeasy extraction kit (Qiagen Inc.) according to the manufacturer’s instructions.

A probe-based qRT-PCR was developed to quantify PRRSv RNA levels in serum and tissue samples. Primers were designed to target a highly conserved region at the C-terminal end of ORF7 of NVSL 97–7895. A dilution series (10^7^ to 10^1^ copies/µl) of *Hin*dIII linearized plasmid, pCR2.1TOPO-NVSL, containing a 446 bp sequence of ORF7 was used as a standard curve. Standards were run in triplicate on each PCR plate and tested samples were run in duplicate on a Stratagene MX3500P (Agilent Technologies, Mississauga, ON). Positive and negative tissue or serum controls for every batch of RNA extraction, and no template controls for every PCR run, were included on each plate. Reactions contained 2 µL of sample or standard, 12.5 µL of Master mix (Brilliant II qRT-PCR Low ROX 1-Step Master Mix, Agilent technologies Canada Inc., Mississauga, ON), 5 pmol of PRRS-2F primer 5′-TAA TGG GCT GGC ATT CCT-3′, 5 pmol of PRRS-1R primer 5′-ACA CGG TCG CCC TAA TTG-3′, 5 pmol of PRRS-P1 probe 5′-HEX-TGT GGT GAA TGG CAC TGA TTG RCA-BHQ2-3′ (reported initially by Kleiboeker *et al.* [21]), 1 µL of reverse transcriptase/RNase block enzyme mixture (Brillant II qRT-PCR Master Mix, Agilent technologies Canada Inc., Mississauga, ON), and RNAse-free water to a total volume of 25 µL. Reverse transcription was performed at 50**°**C for 30 min followed by a PCR initial activation step at 95**°**C for 10 min, and 40 cycles of a two-step denaturation (30 s at 95**°**C) and annealing/extension (30 s at 59**°**C). Assay performance was monitored closely using process behavior control charting. RNA was re-extracted and entire plates re-run if the no template or negative RNA extraction control tested positive by PCR, or if the RNA concentration of the positive control fluctuated above or below the 3-sigma natural process limits on the appropriate control charts. Individual samples were repeated if one of the two replicates was positive but fell below the lowest concentration standard, or if one of the two replicates had no C_t_ value. Over the course of the experiment (approximately 190 plates), the inter-plate coefficient of variation (CV%) for the positive control samples was 11% for serum and 9% for tissues.

### PRRSv ORF5 Sequencing

To detect possible viral mutations in gilts and fetuses compared to the inoculum strain (NLSV 97–7895), the hypervariable region of ORF5 was sequenced. For this purpose, D6 gilt sera and tissues from the fetus in each litter with the highest PRRSv RNA concentration were used. If no PRRSv amplification product could be obtained from fetal sera, RNA was extracted from fetal thymus. Selected RNA samples were first converted into cDNA using the Sensiscript RT kit (Qiagen Inc.) according to the manufacturer’s instructions. The cDNA was amplified using the HotStar Hi-fidelity polymerase kit (Qiagen Inc.) using the following primers: PRRSv-ORF5F: 5′-CCT GAG ACC ATG AGG TGG G-3′ and PRRSv-ORF5R: 5′- TTT AGG GCA TAT ATC ATC ACT GG-3′ [22]. PCR conditions were: inactivation for 5 min at 95**°**C, 35 of cycles of 50 s at 94**°**C, 50 s at 52**°**C, 60 s at 72**°**C, and a final extension step of 10 min at 72**°**C. PCR products (763bp) were confirmed by gel electrophoresis before being purified using the QIAquick PCR purification kit (Qiagen Inc.). Sequencing was performed by Macrogen (Seoul, Korea), analyzed using Staden Package software [23] and aligned with Clustal X2. Sequences from infected animal tissues were compared to the inoculum strain, and the number and location of nucleotide mismatches as well as predicted amino acid changes were determined.

### Evaluation of PPV, PCV2 and TTV

To determine the presence of concurrent infections by PCV2, PPV, and genotype 1 and genotype 2 of swine Torque Teno Virus (TTV g1 and g2) fetal tissues were screened by PCR in a selected number of targeted litters. Twenty-two litters blocked by PRRSv status (9 CTRL, 13 INOC), biweekly rep, presence of mummified fetuses and litter size were selected. All available fetuses were tested from those 22 litters.

From each fetus, DNA was extracted from 10–20 mg of spleen using the DNeasy Blood & Tissue kit (Qiagen Inc.) according to the manufacturer's instructions. PCV2 PCR was performed as described by Gagnon *et al.* [24] using a PCV2 plasmid containing target sequence as a positive control. For PPV screening, a SYBR-green qPCR was performed [25] with DNA extracted from a field case used as a positive control. TTV g1 and g2 were screened using a conventional nested PCR [26] with plasmid containing target sequence as a positive control.

### Statistical Analysis

Descriptive statistics evaluating differences in litter size between INOC and CTRL were performed using a Mann Whitney U test. Treatment group differences in dichotomous outcomes (presence of fever, feed intake reduction, DNA viruses) were evaluated using a Fisher’s exact test. Potential differences in the presence or absence of nucleic acid point mutations and resulting amino acid substitutions between live and dead fetuses were also evaluated using a Fisher’s exact test.

All remaining analyses were performed using two-level, linear (XTMIXED) or logistic (XTMELOGIT) mixed-effects regression models controlling for litter of origin (gilt_id) as a random effect (Stata, StataCorp, College Station, TX). Unless otherwise stated, analyses used data from INOC litters only. Firstly, separate linear univariate models were developed to determine if viral load in fetal thymus, endometrium and fetal serum differed across fetal preservation category (VIA, MEC, DEC, AUT). Logistic regression was used to determine the odds ratio of fetuses from different preservation categories being qRT-PCR positive in fetal thymus, endometrium and fetal serum. Secondly, potential relationships in viral load between various fetal-specific tissues (fetal thymus vs. endometrium, fetal serum vs. endometrium) were compared while accounting for fetal preservation category. Thirdly, the association between fetal body weight of viable fetuses and viral load was compared in two separate analyses: a) using the log (base 10) transformed RNA concentration in fetal thymus, and b) by dichotomizing each fetus as PRRS positive or negative (positive defined as qRT-PCR positive in either fetal thymus or fetal serum or both, negative defined as qRT-PCR negative in fetal thymus and serum). Finally, the body weights of viable fetuses were compared between inoculated and control gilts. Insufficient numbers of MEC, DEC, and AUT fetuses in CTRL litters precluded this analysis for these categories. As above, litter of origin was included as a random effect. These models evaluating effects on fetal weight accounted for total litter size excluding MUM.

When building these models, we considered including biweekly rep as well as litter of origin as random effects. As there was minimal variation at the rep level however, rep was dropped in favour of more parsimonious two-level models (fetuses within gilts). All final models were evaluated to ensure normality and homoscedasticity of residuals. *P*<0.05 was considered statistically significant.

## Results

### Clinical Signs and Litter Outcomes

No gilt demonstrated respiratory signs including dyspnea or persistent paroxysmal coughing, or showed signs of lethargy or depression following challenge. Reduced daily feed intake was observed in 36 of 111 (32%) INOC gilts. In 19 of the 36 gilts, reduced feed intake or complete anorexia was observed for two or more consecutive days. This trended higher in the INOC compared to the CTRL group (*P* = 0.074) in which 3 gilts demonstrated a reduction in feed intake lasting one day each. The presence of fever, defined as a gilt with a rectal temperature exceeding 39.5**°**C any day after PRRSv inoculation was also statistically higher in INOC compared to CTRL gilts (INOC –35/111, CTRL –0/19; *P* = 0.002). Elevated rectal temperatures in INOC gilts showed a biphasic pattern with the first peak occurring on D2 and D3, and the second peak observed between D8 and D10.

One INOC gilt died suddenly on D11. Necropsy examination revealed pulmonary congestion and edema but no interstitial pneumonia. Association with PRRSv infection could not be ruled out. Five of 14 fetuses were decomposed, having died prior to the death of the gilt. Two INOC gilts aborted, one on D17 and one on D20 with 16 and 14 fetuses, respectively. Results from these three gilts were excluded from further analysis.

**Figure 1:**
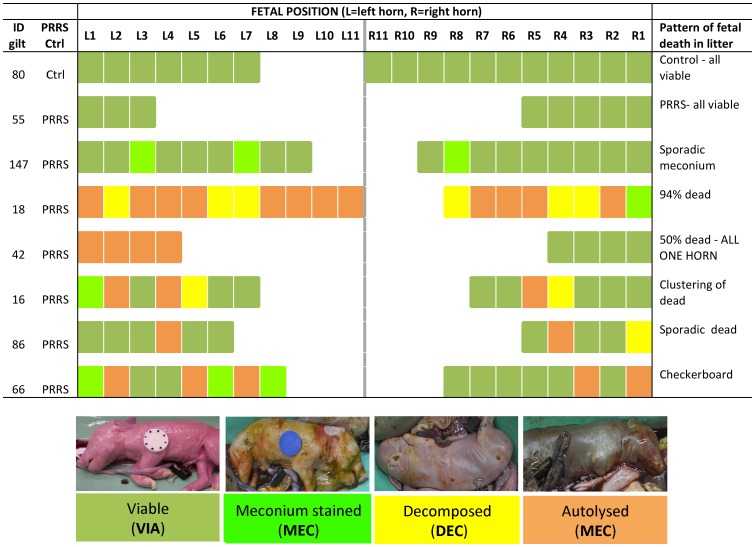
Variation in fetal preservation by position within left and right uterine horns of representative PRRSv inoculated litters at 21 days post inoculation. Fetal preservation categories by location in 8 representative litters are shown (1 CTRL, 7 PRRS). Colours represent the four fetal preservation categories (viable, meconium stained, decomposed and autolysed). Each rectangular cell represents a fetus corresponding to a position: left (L) 1–11, and right (R) 1–11. Gilt identification and PRRS status are provided. CTRL = control, INOC = PRRSv inoculated.

Average litter sizes (excluding mummies) were 12.5±3.7 (INOC, n = 111) and 12.0±3.5 (CTRL, n = 19). Mummified fetuses from INOC (n = 30) and CTRL (n = 4) were excluded from further analysis. Neither average litter size nor number of mummified fetuses differed statistically between INOC and CTRL groups. Percent dead fetuses (DEC+AUT) were significantly higher (*P*<0.01) in INOC (41.0±22.8%) compared to CTRL (1.4±3.4%). In INOC gilts, % of dead fetuses (DEC or AUT) at termination varied from 0% to 94.4%. The position of live and dead fetuses within the litter also varied, with some dead fetuses appearing to cluster in small groupings, with others appearing solitary at random positions. A number of interesting patterns were observed including the complete disparity between left and right horns (Figure 1). In 11 of 111 (9.9%) INOC gilts, fetal survival was 100%. It was apparent from early in the project that meconium stained fetuses were almost exclusively observed in INOC gilts. Thus, we believe that this represents a pathological condition associated with PRRSv infection. Of the 59% of fetuses that survived to termination, about 9% were meconium stained. Although these fetuses were alive at termination, it is probable that many would have died shortly after birth or prior to farrowing if pregnancies were maintained until normal term. A breakdown of fetal preservation categories in inoculated and control gilts is given in Table 2.

**Table 2:** Breakdown of fetal preservation category in PRRSv inoculated (INOC) and reference control (CTRL) litters at 21 days post infection with 1×10^5^ TCID_50_ NVSL 97–7895.

	INOC		CTRL	
	n	%	n	%
VIA	697	50	222	97.8
MEC	125	9	2	0.9
DEC	111	8	0	0.0
AUT	459	33	3	1.3
TOTAL	1392	100	227	100

Number (n) and percentage (%) of fetuses are given for each preservation category (as defined in Table 1) from 111 inoculated and 19 control gilt litters.

### Quantification of PRRSv RNA in Gilt and Fetal Tissues

**Figure 2:**
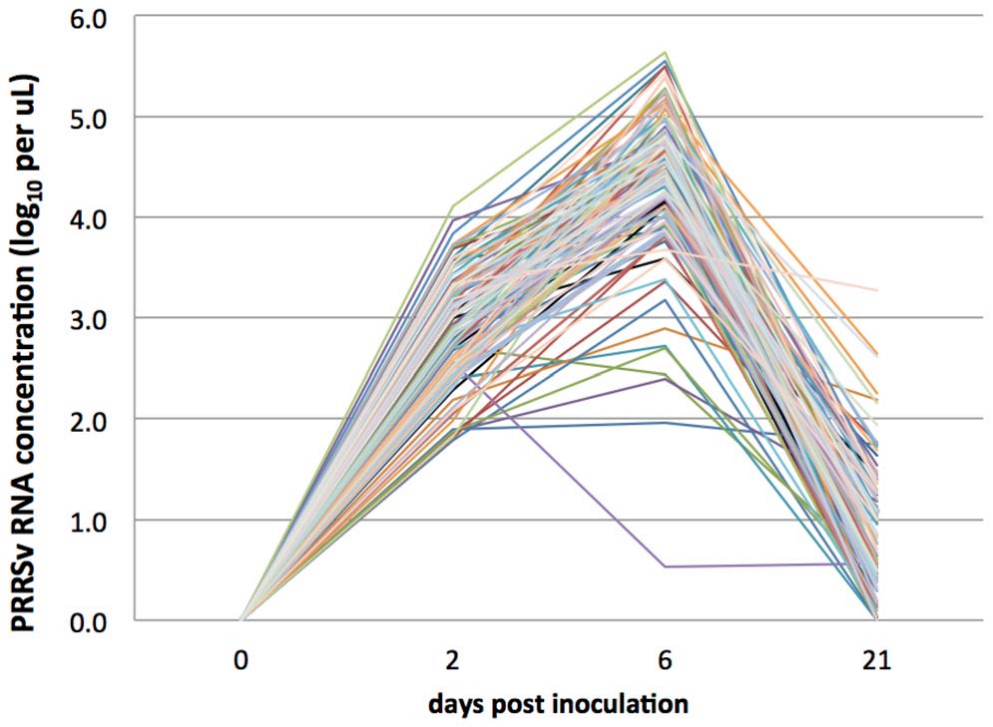
Variation in viremia in PRRSv infected gilts over 21 days post inoculation. RNA concentration (log_10_ target copies/µL) in sera of 111 PRRSv inoculated gilts measured by qRT-PCR.

PRRSv RNA was not detected by qRT-PCR in any tissue or serum sample from CTRL, nor from INOC sera collected prior to inoculation on D0. All INOC gilts were viremic on D2 and D6, and 94 of 111 (84.7%) remained viremic on D21. Peak levels of viremia occurred on D6 for all except two gilts, averaging 4.3±0.8 log10 copies/µL (range 0.5 to 5.6) (Figure 2). Nearly all INOC tonsil and lymph nodes tested positive by qRT-PCR at termination. The highest viral loads in gilt tissues were found in reproductive lymph node (*Lnn. uterini*), which tested positive in 100% of inoculated gilts (Table 3). By contrast, the lowest viral loads and positive prevalence were found in lung.

**Table 3:** Concentration of PRRSv RNA in tissues and serum of PRRSv inoculated gilts and their fetuses (log_10_ RNA copies/µL or mg).

Tissue/Serum	Number tested	% positive	Number positive	Mean log_10_ copies/µL or mg	SD
Serum 2 dpi	111	100	111	2.9	0.5
Serum 6 dpi	111	100	111	4.3	0.8
Serum 21 dpi	111	84.7	94	0.9	0.6
Lung	111	90.1	100	3.5	1.2
Tonsil	111	99.1	110	5.6	0.8
Reproductive LN	111	100	111	5.8	0.8
Tracheobronchial LN	111	99.1	110	4.8	0.9
Fetal thymus§	1391	72.8	1013	4.7	2.0
Endometrium*	1392	85.1	1185	3.9	1.7
Fetal serum§	840	67.1	564	4.3	2.8

RNA concentration is presented as mean log_10_ copies/µL of serum or mg of tissue from qRT-PCR-positive samples.

SD = standard deviation (SD), LN = lymph node, % positive = percentage of samples tested positive within each category.

§ The thymus sample from one autolysed fetus and serum samples from two viable fetuses were missed during sample collection.

*Endometrium sample collected adjacent to the umbilical stump of each fetus.

**Figure 3:**
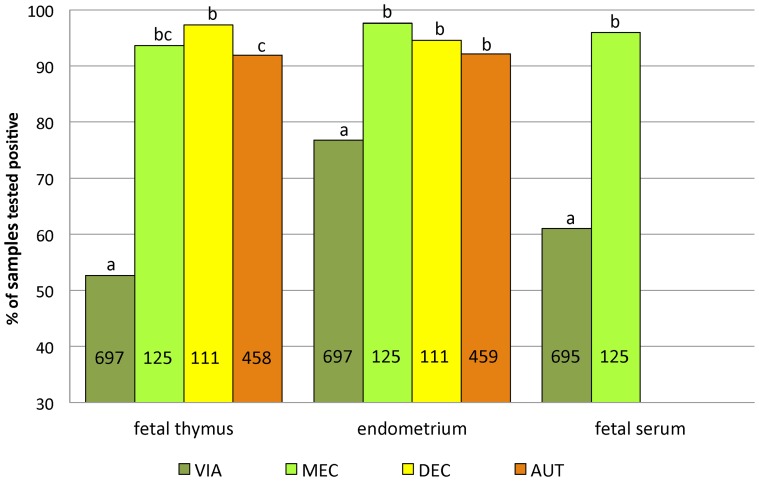
Percentage of fetal samples testing positive by qRT-PCR by preservation category. Percentage of thymus, endometrium, and serum samples positive by qRT-PCR from fetuses of different preservation categories. Values indicate total number of samples tested within each category and sample type. Statistically significant differences in the % positive samples from fetuses of different preservation categories within a tissue type are denoted using different superscript letters (*P*<0.05).

**Figure 4:**
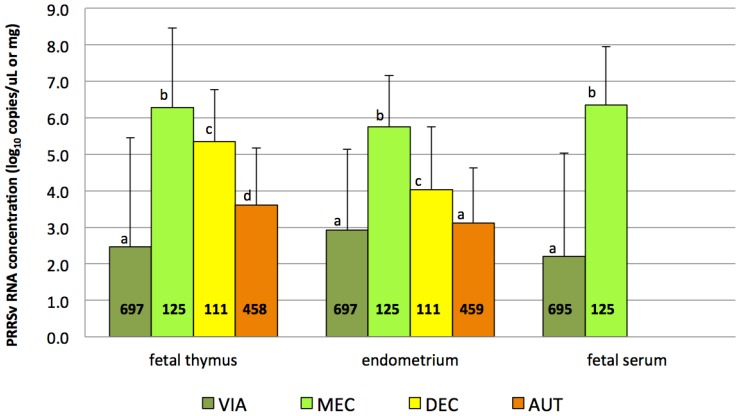
Mean viral load in fetal thymus, endometrium and fetal serum by fetal preservation category. Mean viral load (log_10_ copies/µL of serum or mg of tissue) in fetal thymus, endometrium and fetal serum is presented for different fetal preservation categories from inoculated gilts. Superscript letters indicate significant differences in mean viral load for each preservation category within particular sample type obtained by the linear-mixed models. Values indicate total number of samples tested within each category and sample type. Error bars represent standard deviation. Fetal thymus: a:b, a:c, a:d, c:d, a:d *P*<0.001; b:c *P* = 0.0046. Endometrium: a:b, a:c, b:c *P*<0.001. Fetal serum: a:b *P*<0.001.

All samples from CTRL litters yielded negative results by qRT-PCR, including the CRTL fetuses that were MEC and AUT (n = 5). Quantitative RT-PCR results for fetal thymus, endometrium (from viable and dead fetuses) and fetal serum (from viable fetuses) from INOC gilts are presented in Table 3. Comparison across preservation category are presented in Figure 3 and Figure 4. The odds ratio (*OR*) of a fetus being qRT-PCR positive in fetal thymus was greatest in DEC fetuses (*OR* 37.5 compared to VIA fetuses), while the odds of being qRT-PCR positive in endometrium was greatest in MEC fetuses (*OR* 11.6 compared to VIA fetuses). MEC fetuses were 15.8 times more likely to be qRT-PCR positive in fetal serum than VIA fetuses (*P*<0.05 for all).

PRRSv RNA concentration in fetal thymus, serum and endometrium differed significantly across preservation categories (Figure 4). In all three tissues, lowest viral loads were measured in VIA fetuses. Mean RNA concentrations were significantly higher in MEC fetuses than in fetuses from all other preservation categories. Viral loads in fetal thymus and endometrium of DEC fetuses were significantly lower than those of MEC fetuses, but significantly higher than those of AUT fetuses (*P*-values see Figure 4). Viral loads in fetal thymus and in fetal serum were positively associated with viral load in endometrium. A one-log_10_ increase in PRRSv RNA concentration in endometrium was associated with an increase of 0.66 log_10_ RNA copies/mg fetal thymus (*P*<0.001) and 0.87 log_10_ RNA copies/µL fetal serum (*P*<0.001), respectively.

A total of 215 (of 840) fetuses were negative by qRT-PCR in both thymus and serum, while virus could be detected in the associated endometrial sample. Most of those fetuses (210 of 215) were viable. Similar results were observed when comparing qRT-PCR results from fetal thymus and endometrium, which were available for all fetuses; 378 fetuses were qRT-PCR negative in thymus but positive in endometrium, and 330 of these were viable.

### PRRSv Susceptibility

**Figure 5:**
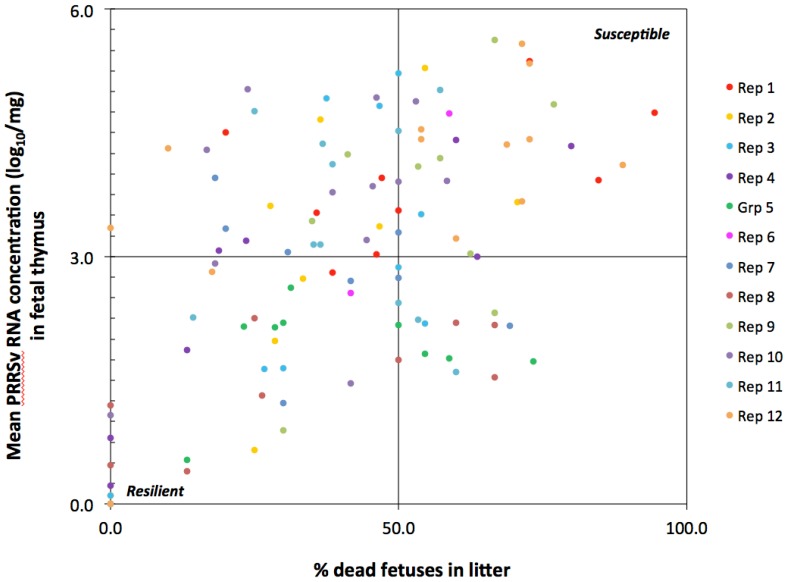
Variation in PRRSv litter susceptibility. Each dot represents a single PRRSv infected litter, positioned on X-Y grid based on percentage of dead fetuses in litter (X-axis) and mean PRRSv RNA concentration in the thymus of all live and dead fetuses in the litter. Susceptible outcomes (top right quadrant) are litters with high percent dead and high viral loads in fetal thymus. Resilient litters (bottom left) are those with low percent dead and low or negative viral loads in fetal thymus.

A two by two chart comparing percent dead fetuses and mean viral RNA concentration in fetal thymus was developed to visualize the variation in susceptibility among INOC litters (Figure 5). Susceptible outcomes were classified as litters with a high percentage (>50%) of dead fetuses (DEC, AUT) and high PRRSv RNA viral loads (>3.0 log_10_ copies/mg) in thymus of all fetuses in the litter. Approximately 30 of 111 gilts across multiple reps fell into this category. By contrast, PRRSv resilient litters, a highly desirable trait, were those with a low percentage (<50%) of fetuses dead and low or negative PRRSv RNA levels (<3.0 log_10_ copies/mg) in fetal thymus. Approximately 17 gilts across multiple reps fell into this category.

### PRRSv ORF5 Sequencing Results in Gilts and Fetuses

DNA sequencing of ORF5 PCR products amplified from gilt sera collected 6 dpi showed 100% agreement with the sequence of the inoculum strain in 106 of 111 INOC gilts. For 5 gilts, a single nucleotide change was observed. In two of these, the nucleotide change did not result in an amino acid change. In the remaining three gilts, however, a nucleotide substitution from thymine (T) to cytosine (C) was detected at position 304 of ORF5 resulting in an amino acid change from tyrosine to histidine (Table 4). Interestingly, identical changes were observed in the tested fetus in 2 of these 5 gilts.

**Table 4:** Nucleotide position, nucleic acid and amino acid changes in ORF5 sequences in serum and tissues of INOC gilts and fetuses.

Nucleotide position in ORF5	Consensus nucleotide change	Amino acidchange	Gilts affected(serum 6 dpi)	Fetuses affected(fetal serum or thymus)
32	G to A	C to Y		G62_L3
36	G to A	nil		G70_R2
80	C to T	A to V		G27_L3, G41_R5, G146_R3
94	A to G	N to D		G142_L8
95	A to G	N to S		G16_L6, G81_L4, G97_L6, G141_R4
132	C to T	nil	G54	
139	C to T	nil		G30_R3, G70_L4, G70_R2, G83_R3, G102_L4, G142_L8
169	A to G	K to E		G81_L4
170	A to G	K to R		G141_R4
173	A to G	D to G		G30_R3
304	T to C	Y to H	G56, G71, G101	G56_L3, G57_R5, G71_R6
363	T to C	nil		G86_L3
366	C to T	nil		G139_R3
369	C to T	nil		G121_L5
432	C to T	nil		G85_L7
435	T to C	nil	G138	G138_L2
436	C to T	nil		G116_L5
475	G to A	V to I		G95_L7
480	C to T	nil		G85_L7
507	C to T	nil		G81_L4

Nucleic acid abbreviations: A = Adenine, C = Cytosine, G = Guanine, T = Thymine.

Amino acid abbreviations: A = Alanine, C = Cysteine, D = Aspartic acid, E = Glutamic acid, G = Glycine, H = Histidine, I = Isoleucine, K = Lysine, N = Asparagine, R = Arginine, S = Serine, V = Valine, Y = Tyrosine.

No nucleic acid substitutions were detected in 106 of 111 INOC gilt sera samples, nor in 76 of 99 fetal thymus samples. ORF 5 sequences were not obtained in 12 of 111 fetuses.

In 12 of 111 PRRSv-challenged litters, high quality cDNA could not be obtained in either fetal thymus or serum to enable ORF5 sequencing. Therefore, ORF5 sequences were obtained and analysed for one fetus in each of 99 INOC litters. The obtained sequence was identical to ORF5 from NVSL97–7895 in 76 of 99 (77%) fetuses. For 17 fetuses, a single nucleotide change was detected which in 10 cases resulted in an amino acid change (Table 4). Two nucleotide changes were found in sequences from 4 fetuses resulting in two, one and no amino acid substitutions in one, two and one fetus respectively. The sequence from one fetus showed 3 nucleotide mismatches; two of them resulted in amino acid changes (Table 4). The ORF5 sequence from one fetal sample (G70_R5) resulted in a total mismatch compared to the inoculation strain. Therefore, cDNA sequencing from the same fetus and from two additional fetuses of the same litter (G70_L4, G70_R2) was repeated. Sequences obtained showed zero, one and two nucleotide mismatches, respectively (Table 4).

Nucleotide substitutions were located at over 20 positions in ORF 5. At four positions the same substitution was observed in multiple animals (positions 80, 95, 139, 304). A point mutation at position 139 was most frequently observed (6 fetuses in 5 litters) but did not result in an amino acid change. By contrast, the point mutations at positions 80, 95 and 304 in multiple animals did result in amino acid changes. However, neither nucleotide nor single or multiple amino acid substitutions, were associated with fetal preservation category in the present study (*P*>0.05).

### Fetal Weight

**Figure 6:**
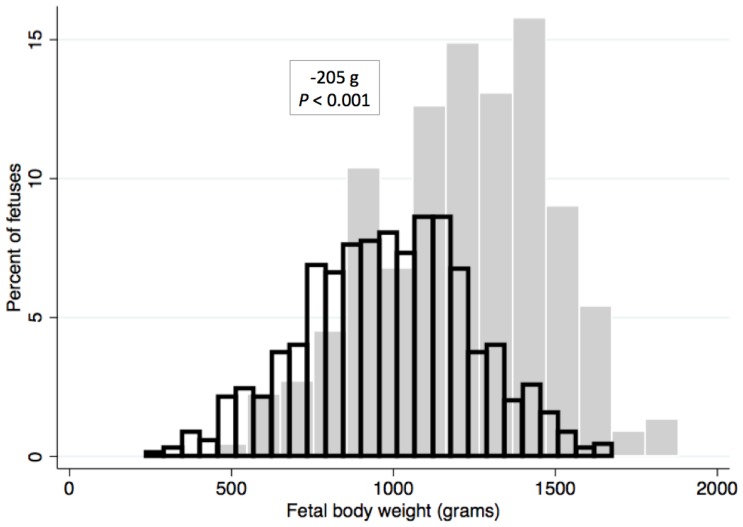
Distribution of viable fetus body weights of PRRSv-infected and control litters. Weights were measured 21 days post inoculation at gestation day 106 (±1) in 111 PRRSv infected (black outline bars) and 19 control litters (grey bars). INOC: 999.3±21.1 g, CTRL: 1204.1±36.4 g, after accounting for litter of origin and litter size (*P*<0.001).

The body weight of viable fetuses from INOC (999.3±21.1 (SE) grams) was significantly lower than from CTRL fetuses (1204.1±36.4 g) after accounting for litter of origin (*P*<0.001) (Figure 6). No association could be detected between body weight of viable fetuses and viral load in fetal thymus (*P* = 0.91) nor between fetal weight and PRRSv positivity of the fetus (*P* = 0.93), after accounting for litter size.

### DNA Virus Screening

In total, 261 fetuses from 9 CTRL and 13 INOC gilts were screened for PCV2, PPV and TTV g1 & g2. Sporadic positive results, equally spread among all 12 reps, were obtained across litters from both groups. PCV2 was detected in 17 fetuses from 12 litters (5 CTRL, 7 INOC) and 16 of those fetuses were weakly positive. No more than 2 fetuses were positive for PCV2 within any litter. In three litters (2 CTRL, 1 INOC), one fetus was weakly positive for PPV. TTV g1 was detected in 5 fetuses, in one fetus in a CTRL and in four fetuses in one INOC litter. TTV g2 was detected in 23 fetuses from 11 litters (5 CTRL, 6 INOC); 18 showed a weak band on the gel and were therefore considered weakly positive. Detection frequency did not differ statistically between INOC and CTRL for any of these 4 DNA viruses. Furthermore, the presence of the DNA viruses did not differ statistically amongst fetal preservation categories in INOC litters.

## Discussion

The virus strain used in the present study was reported to be of high virulence, causing cases of exacerbated reproductive failure in the Midwest of the United States in the mid-1990 s [20]. No severe clinical signs were observed in our gilts following infection, although all inoculated gilts became infected. QRT-PCR results from gilt sera and tissues are in accordance with previous reports which showed that virus can be found in lymphatic tissues after it was cleared from the blood [27]. The infection of gilts on gestation day 85 (±1) resulted in the death of ∼41% of fetuses with a very high level of variation between litters. Targeted screening for DNA viruses supports the conclusion that co-infections with PCV2, PPV and TTV g1 & g2 did not influence the results of the study.

About 9% of fetuses from inoculated gilts were categorized as meconium-stained. Those fetuses were PRRS affected but alive on the day of termination. However, the amniotic fluid was inspissated, yellow-brown in colour, decreased in volume, and the umbilical cord of many of those fetuses was edematous. Although meconium-staining results from fetal stress at the time of parturition, we believe the meconium-staining observed in this study was most likely a pathologic indicator of PRRSv infection, since meconium-stained fetuses were only observed in PRRS-challenged litters with the exception of two fetuses from one CTRL litter. Considering that the study was terminated on gestation day 106±1, we believe the meconium-stained fetuses would most likely have been born dead if gilts in this study farrowed naturally. We therefore predict that at least 50% of fetuses from inoculated gilts were affected by PRRSv in this study.

The clinical effects of reproductive PRRSv however are variable, with clinical outcome dependent on the viral strain, host and environmental factors. Approximately 40% (18 of 46) of fetuses from dams inoculated with type 2 PRRSv (strain SD-23983) on gestation day 90 showed some form of gross pathologic change when terminated at 19–22 dpi, but only 2 of 46 fetuses were found dead in that experiment [14]. By contrast, 75% and 74% of piglets, respectively, were stillborn from sows and gilts experimentally inoculated with either type 2 or type 1 PRRSv 3 weeks prior to farrowing [13], [18]. This present study was undertaken to investigate host factors associated with variability in reproductive outcome. In order to enable such an investigation, a large number of gilts were inoculated with the same virus isolate at the same inoculation dose. Our results demonstrate that the outcome of infection is extremely variable. Ongoing analyses will determine if phenotypic or genotypic factors affect susceptibility to PRRSv infection at the level of the dam or fetus.

For reproductive PRRS, a desirable litter outcome is one with a high number of vigorous, non-infected piglets. Although in the present study approximately 15% of fetuses were categorized as viable and had no virus detected in thymus or serum, there was only one INOC litter in which all fetuses were viable and free of virus based on qRT-PCR. To increase confidence in these results, qRT-PCR was repeated in fetal thymus, serum and spleen and all results were confirmed negative. Despite being a highly valued trait, complete resistance to PRRSv infection is a very rare event. That being said, there was clearly a wide range in susceptibility in gilts and fetuses in this trial. There are a number of ways to determine PRRSv susceptibility in animal models and the simple 2×2 chart used herein to display percent dead by fetal thymus levels helped to visualize the variation in susceptibility. Until the mechanisms of fetal death are more clearly understood, charts such as this can be further refined to reflect the most biologically relevant phenotypes.

Meconium-stained fetuses showed significantly higher viral loads in endometrium, fetal thymus and serum compared to fetuses from other preservation categories. Viral levels were significantly lower in decomposed and even lower in autolysed fetuses, likely due to viral RNA degradation over time. Viable fetuses, of which approximately 50% tested negative by qRT-PCR, had the lowest viral levels and positive prevalence but demonstrated tremendous variability. Therefore, meconium-stained fetuses appear to be the most suitable for routine diagnostics, followed by decomposed (recently dead) fetuses. In contrast, Han *et al.* [18] infected pregnant gilts with a type 1 PRRSv isolate but did not find significant differences in the number of genomic copies between stillborn and viable fetuses on the day of farrowing. Differences in the virulence of the PRRSv used as inoculum and larger sample sizes and experimental power in the present study may explain these contradictory results.

Viral levels in endometrium adjacent to each fetus were strongly associated with levels in fetal thymus and serum. However, virus was detected more frequently in endometrial samples than in either fetal sera or thymus. The mechanisms of fetal death are still poorly understood. It was previously reported that viral replication in fetal implantation sites and associated apoptosis of infected macrophages and surrounding cells precedes fetal infection and might be responsible for the induction of reproductive disorders [11], [18]. According to our results, the presence of detectable PRRSv RNA in endometrium by qRT-PCR did not necessarily result in fetal death. However, fetuses that were qRT-PCR positive in the surrounding endometrium but negative in fetal serum and/or thymus could represent cases of early infection, before the virus was able to transmit from the dam to the fetus. Interestingly, viral levels in endometrium of fetuses that were qRT-PCR negative were comparatively low, with mean values less than 2 log_10_ copies/mg tissue. A minimum concentration of virus may be necessary for the virus to cross the placental barrier and infect the fetus.

PRRSv isolates show a remarkable degree of genetic variability. This influences virulence, the development of protective immune responses, and possibly persistence of infection. It has been demonstrated that the PRRSv evolves continuously during replication in pigs, with ORF5 being the most diverse region of the PRRSv genome [28], [29]. In accordance with previous studies [28], [29], the nucleotide substitutions detected in ORF5 sequences from gilts and fetuses appeared to be random and the overall mutation rate was low. However, nucleotide changes were not predominantly found among amino acid residues near the N terminus or C terminus, but were detected in the more conserved, central regions of ORF5 [28]–[31]. The significance of mutations in ORF5 is not completely understood, but it has been shown that a single genotype can become dominant due to increased viral fitness [28], [29].

Fetal infection in pregnant sows and gilts might present an additional source of PRRSv diversity since fetuses are capable of selecting for a particular virus population [14]. While there is tremendous opportunity to investigate the ecology of PRRSv diversity in this reproductive dataset, it is beyond the scope of this project. That being said, the most frequent point mutation in the present study occurred at position 139 of ORF 5. Since it did not result in an amino acid substitution, it would not likely have altered the fitness of the virus. By contrast, point mutations at positions 80, 95 and 304, noted in multiple animals, did result in amino acid substitutions. While the possibility of fitness changes cannot be ruled out and it would be speculative to conclude anything based on a small sample size, the biological significance of these substitutions is worthy of further investigation.

Viable fetuses from PRRSV-infected gilts weighed less than those from control gilts. Since fetal weight was influenced by preservation category and almost all fetuses from control gilts were viable, the analysis was performed for viable fetuses only. For growing pigs, body weight gain is negatively associated with viral load [32]. However, in our study we could not associate viral loads in fetal thymus, serum or endometrium with fetal weight. This could be explained by the fact that both viral load and fetal weight were measured on D21, and it is not possible to measure weight gain or viremia in fetuses over the course of infection as in growing pigs.

In conclusion, in this large scale study we have demonstrated that the extent of disease in fetuses from type 2 PRRSv-infected gilts was highly variable, both between gilts and often within individual litters. We also determined that meconium-staining of fetuses is an early pathological condition of reproductive PRRS. Meconium-stained fetuses had the highest levels of virus in tissues. Although mechanisms of fetal death are not clearly understood, viral load in endometrium was positively associated with viral load in fetal thymus and serum indicating that the virus exploits dynamic linkages between individual maternal-fetal compartments. Even though point mutations in ORF5 were not related to fetal outcome and were most often randomly located, there were 4 positions with more frequent mutations; three of these were associated with amino acid substitutions. Finally, we estimate PRRSv infection decreased the weight of surviving fetuses by approximately 17%. In future work, more detailed phenotypic responses of gilts and fetuses will be investigated to determine potential mechanisms and host genetic variation associated with fetal death.
